# Supplementary material for: Neurogenetic phenotypes of learning-dependent plasticity for improved perceptual decisions
Source: Commun Biol. 2025 May 21;8:779. doi: 10.1038/s42003-025-08212-7 (PMC12095785; doi:10.1038/s42003-025-08212-7)
Supplement: Supplementary file 2 — Supplementary Information [file 42003_2025_8212_MOESM2_ESM.pdf]

Supplementary material

Participants

For the multi-session training study, only data from male participants was included. The data was collected as part of a previous study, which included MRS data collection (Ziminski et al., 2023). The study sample was limited to male participants due to potential confounding effects of menstrual cycle on GABA measurements. This has been the topic of extensive research with several studies restricting MRS-GABA studies to males. Key hormones (estrogen, progesterone) exerting a suppressive or facilitatory effect on GABA transmission may confound within-subject GABA measurements over time. Developing precise methods for controlling for the effects of menstrual cycle on MRS GABA measurements is hampered by physiological complexity (i.e., phase and regional effects of menstrual cycle on GABA) and limited knowledge of the kinetics of menstrual cycle GABA changes in humans. As the study involves repeated MRS GABA measurements over time, it is not possible to satisfactorily control for menstrual cycle effects; i.e., not only the phase and duration of the menstrual cycle but also the kinetics of GABA changes across menstrual cycle days would likely differ substantially across participants.

For the tDCS and training intervention study, data was collected from both male and female participants.

Stimuli

Participants were trained to distinguish radial versus concentric Glass patterns (Glass, 1969) embedded in noise (SN task; Figure 1A). Stimuli (size = 7.9˚ × 7.9˚) comprised of white dot pairs (dipoles) that were presented within a square aperture on a black background. The stimulus parameters followed previous studies (Frangou et al., 2018, 2019). The dot density was 3%, and the Glass shift (i.e., the distance between 2 dots in a dipole) was 16.2 arc min. The size of each dot was 2.3 × 2.3 arc min^2. Radial and concentric patterns were generated by

placing dipoles orthogonally (radial) or tangentially (concentric) to the circumference of a circle centered on the fixation dot. The spiral angle was defined as the angle between the dot dipole orientation and the radius from the dipole center to the center of the stimulus aperture. For radial patterns the spiral angle was 0˚ and for concentric 90˚. Each stimulus consisted of dot dipoles aligned according to either the radial or concentric spiral angle and noise dipoles for which the spiral angle was randomly selected. The ratio of signal to noise dipoles defined the stimulus signal level; stimuli were presented at 24% ± 1% signal level; i.e., 76% of the dipoles were presented at random position and orientation based on (Mayhew et al., 2012).

We randomized the presentation of clockwise (0˚ to 90˚ spiral angle) and counterclockwise patterns (0˚ to −90˚ spiral angle) across participants. To control for potential local adaptation and ensure that learning related to global shape rather than local stimulus features, we generated a new pattern per trial and jittered (±1 to 3˚) the spiral angle across stimuli. Stimuli were presented at the left hemi-field (11.6 arc min from fixation) similar to our previous studies(Frangou et al., 2018).

Multi-session training study: Behavioral session design

All behavioral sessions took place in the lab (Ziminski et al., 2023). Each volunteer participated in six sessions: three brain-imaging sessions including testing on the SN task without feedback (day 1: baseline, day 5: pre-training, day 9: post-training) and three consecutive task training behavioral sessions with feedback (day 6, day 7, day 8). Baseline, pre-training, and post-training followed the same paradigm design. During each session, participants completed 8 runs (200 trials per run) of the SN tasks. Each run comprised an equal number of trials presenting concentric and radial patterns. In each trial, a fixation dot appeared for 500-1500ms, followed by a radial or concentric pattern for 300 ms. Participants were asked to judge whether the stimulus presented was radial or concentric. Trial-by-trial feedback was provided by means of a visual cue (green tick for correct, red “x” for incorrect), which remained on the screen for

200 ms and was followed by a fixation dot for a variable time between 500 and 1,500 ms before the next trial onset. Data from 2 participants were excluded because they did not show improvement in the behavioral task, as defined by a positive learning rate across sessions (from baseline to post-training).

Multi-session training study: Behavioral data analysis

To quantify discriminability between the two Glass patterns classes (radial vs. concentric), we computed d' (Stanislaw & Todorov, 1999) across trials per run, as the difference between the z-transform of each stimulus class' hit and false alarm rates. We calculated a) performance accuracy (*i.e.* percentage of correct responses) for each session, b) learning rate that indicates the rate of change in perceptual sensitivity as measured by d' per training run. To compute learning rate, we fitted individual participant training data with a logarithmic function: $y = k * \ln x + c$, where x is the training run, y is the run d', c is the starting d' and k corresponds to the learning rate, using MATLAB 2013a (The MathWorks, Natick, MA, USA). Data of two participants were excluded from further analyses as they had a negative learning rate.

Multi-session training study: MRI acquisition

MPM data acquisition. Whole-brain MPM data were collected using a spoiled multi-echo 3D fast low-angle shot (FLASH) protocol (Callaghan et al., 2019) of three gradient acquisitions: magnetisation transfer (MT) saturation, T1-weighted and proton density (PD) weighted maps. We used a semi-quantitative MT saturation (MTsat) sequence to account for spatially varying T1 and B1+ and to enhance specificity to myelin content (Helms, Dathe, Kallenberg, et al., 2008; Tabelow et al., 2019). We also collected B0 fieldmap and B1 fieldmap to correct for field inhomogeneities and susceptibility distortions. MPM data were acquired while participants watched a neutral nature documentary (Dynasties; BBC).

Res-fMRI data acquisition. Echo-planar imagining (EPI) acquisitions with full brain coverage (TR: 727 ms, TE: 34.6 ms, slices: 72; voxel size: 2 mm isotropic; multi-band factor: 8; flip

angle 48°; volumes: 812) were collected. Resting-state fMRI data were acquired while participants fixated on a central white cross presented on black background. Cardiac and respiratory signals were recorded using a pulse oximeter and respiratory belt respectively.

Multi-session training study: MRI data analysis

MPM maps. We used processed MPM maps from our previous study (Ziminski et al., 2023). In brief, T1w images from three MRI sessions (1st echo) were segmented in SPM and brain-masked prior to longitudinal registration with the CAT12 toolbox (http://www.neuro.uni-jena.de/cat/). For each participant, we generated a participant-average T1w image and co-registered the brain-masked T1w images from three MRI sessions to the average T1w image. We applied the same participant-wise transformation to other MPM images (*i.e.* PDw and MTw) from the same session. Then, we used the hMRI toolbox (Tabelow et al., 2019) in MATLAB SPM 12 (v7771; Wellcome Centre for Human Neuroimaging, London, UK), auto-reorient module to reorient the images to anterior commissure with the 1st-session T1 as an individual reference. We used the hMRI toolbox map creation module to generate bias-corrected R1, R2, MT and PD maps. MPM data of five participants were excluded due to poor map quality (PD map error estimate less than 8%, mean / SD white matter intensity) and visual inspection. We then used the MT and R1 maps for microstructural gradient generation.

Parcellation approach. We used a wole-brain parcellation approach (Schaefer et al., 2018) (200 cortical parcels) that combines local gradient and global similarity approaches via gradient-weighted Markov Random models. The parcellation achieved good connectional homogeneity. Each node was then assigned to one of seven canonical functional brain networks (Yeo et al., 2011) with the maps_and_parcs toolbox (Bethlehem & Kitzbichler, 2023).

Microstructural profile covariance (MPC) and microstructural gradients. First, we used FreeSurfer (V6.0.0; http://surfer.nmr.mgh.harvard.edu/) recon-all command to perform cortical reconstruction and volumetric segmentation for MPM R1 maps, resulting in a T1 image

in the native processing space (T1-nativepro). Next, we performed within-subject registration using a boundary-base cost function to register MT images to T1-nativepro images with FreeSurfer bbregister toolbox (Greve & Fischl, 2009). We then used the micapipe toolbox V0.1.2 (Cruces et al., 2022) post_structural module to parcellate T1-nativepro images into Schäefer-200 cortical parcels (Schaefer et al., 2018) and the MPC module to generate the microstructural profile covariance (MPC) matrix for cortical myelination densities based on the equivolumetric surface construction techniques (Waehnert et al., 2014; Wagstyl et al., 2018). Specifically, the equivolumetric surface construction technique generates surfaces with a constant distance fraction from the segmented boundaries (Waehnert et al., 2014) (Fig 2, Eq. (4)-(10)). For each participant per session, we generated a 200 × 200 MPC matrix.

To build MPC gradients, we used the BrainSpace package (Vos de Wael et al., 2020) in Python. First, we computed the mean MPC of the MPCs from MRI session 1 (baseline) and MRI session 2 (pre-training) from all participants as an MPC template. Then, we computed a group gradient template with the MPC template (kernel = normalized_angle, dimensionality reduction approach = diffusion mapping, sparsity = 0.8). We generated and aligned within-subject gradients to the template gradients individually (alignment = procrustes). We extracted the first 10 principal gradients for each participant per session for further analysis.

Resting-state fMRI and functional connectivity (FC) gradients. Resting-state fMRI data were processed with the micapipe toolbox V0.1.2 (Cruces et al., 2022) *-proc_func* module. The module pipeline is based on FSL (Jenkinson et al., 2012) (https://fsl.fmrib.ox.ac.uk/fsl/fslwiki) and AFNI (Cox, 1996) (https://www.opensourceimaging.org/project/afni/) for volumetric processing and FreeSurfer (Fischl, 2012) and Workbench (Marcus et al., 2011) for surface-based mapping. In brief, after distortion correction and motion correction, with high-pass filtering, Multivariate Exploratory Linear Optimized Decomposition into Independent Components (MELODIC) was performed on the filtered timeseries. After registrations (both

linear and non-linear) between fMRI and T1-nativepro space were performed, as well as a boundary-based registration between fMRI and native Freesurfer space, processed timeseries would be registered to native cortical surface (Schäefer-200 parcellation). One participant was excluded from resting-state analysis due to high head movement (> 2 mm). For each participant per session, a 200 × 200 weighted adjacency matrix encoding the connectome was constructed. We used the BrainSpace package (Vos de Wael et al., 2020) to build FC gradients. First, we computed the mean FC matrix of FC matrices from MRI session 1 (baseline) and MRI session 2 (pre-training) as an FC template. Next, we computed a group gradient template with the FC template (kernel = normalized_angle, dimensionality reduction approach = diffusion mapping, sparsity = 0.8). We generated and aligned within-subject gradients to the template gradients individually (alignment = procrustes) for each session. We extracted the first 10 principal gradients for each participant per session for further analysis.

Principal gradient dispersion. To investigate the organisation of microstructural and functional communities within a multi-dimensional connectivity space, we generated the gradient (Bethlehem et al., 2020; Cross et al., 2021) of two established functional networks from Yeo7 networks (*i.e.* the visual network and the frontoparietal network based on gene expression analysis) for both microstructural principal gradients and FC principal gradients bounded by three principal gradients following previous work (Bethlehem et al., 2020). We calculated the sum squared Euclidean distance of network nodes to the network centroid at individual level to get within-network dispersion and the Euclidean distance between network centroids as between-network dispersion. These metrics were calculated for each subject within the individualised, aligned gradient space. Small within-network dispersion value indicates a highly coherent network, while high between-network dispersion indicates networks that are segregated from each other.

tDCS and training intervention study: MRI acquisition

MPM data acquisition. Whole-brain multi-parameter mapping (MPM) data were collected using a multi-echo, fast-low-angle-shot (FLASH) protocol. The MPM FLASH protocol consisted of three gradient acquisitions: magnetisation transfer (MT) saturation (6 echoes: from 2.31 to 14.01ms in increments of 2.34ms, TR = 23.64ms, excitation flip angle of 6°), T1-weighted (6 echoes: from 2.31 to 14.01ms in increments of 2.34ms, TR = 19.17ms, excitation flip angle of 21°), and proton density (PD) weighted maps (8 echoes: from 2.31 to 18.69ms in increments of 2.34ms, TR = 23.64ms, excitation flip angle of 6°). All weighted maps had 1mm isotropic resolution, field of view of 256x240x176mm, readout bandwidth of 465Hz/pixel and were collected with partially parallel imaging (GRAPPA, 18 reference lines, acceleration factor of two). For correcting field inhomogeneities and susceptibility distortions, we further collected B1 (11 measurements, TR = 500ms, TE1 = 39.06ms, TE2 = 16.53ms, 4mm isotropic resolution) and B0 fieldmaps (magnitude-phase, TR = 1020ms, TE1 = 10ms, TE2 = 12.46ms, 3x3x2mm resolution, flip angle of 90°).

Res-fMRI data acquisition. Echo-planar imaging (EPI) data (gradient echo-pulse sequences) were acquired during rest (TR = 727ms; TE = 34.6ms; number of slices = 72; voxel size = 2mm isotropic; Multi-band factor = 8; flip angle = 51°; number of volumes = 660; duration = 8m09s; whole brain coverage). During EPI data acquisition, we recorded cardiac pulsation (using a pulse oximeter) and respiration (using a respiratory belt) to model these physiological data for denoising. Cardiac and respiratory signals were recorded using a pulse oximeter and respiratory belt, respectively. Data from 8 participants (2 Anodal, 6 Sham) were excluded from further analysis due to missing the second rs-fMRI run due to technical problems.

tDCS data acquisition. We used a multi-channel transcranial electrical stimulator (neuroConn DC-STIMULATOR MC, Ilmenau, Germany) to deliver anodal or sham stimulation following our previous study (Frangou et al., 2018). We used a pair of MR-compatible rubber electrodes (3x3 cm^2 stimulating electrode, 5x5 cm2 reference electrode), which were secured on the head

with the help of rubber bands. Ten-20 paste was used as a conductive medium between the rubber electrodes and the scalp. For the Anodal group, 1mA current was ramped up over 10s, was held at 1mA for 20min and was subsequently ramped down over 10s. For the Sham group, the current ramped up (10s) and down (10s) in 518 the beginning of the session. We used online stimulation (i.e. stimulation during training), as this protocol has been previously shown to enhance the lasting effect of training (O'Shea et al., 2017). It has been shown that this facilitatory effect is not present or polarity-specific when stimulation precedes training, with anodal stimulation impeding learning (Stagg et al., 2011). To achieve consistent electrode placement across participants when targeting the right posterior OCT, we placed the bottom right corner of the square stimulating electrode on T6, using a 10-20 system EEG cap, maintaining the same orientation across participants, parallel to the line connecting T6 and O2. The reference electrode was placed on Cz. We have previously used the same electrode montage (Frangou et al., 2018), following electrical field density simulations showing that this montage results in unilaterally localised current density, the peak of the electric field density being under the anode electrode around the posterior.

tDCS and training intervention study: MRI data analysis

MPM maps. MPM data were pre-processed using the hMRI toolbox (Tabelow et al., 2019) in SPM12.4 (v7219; www.fil.ion.ucl.ac.uk/spm/software/spm12/). A T1-weighted template was generated by coregistering the first echo of the two T1-weighted scans to the midpoint and averaging them (Thomas & Baker, 2013). The MT-, T1- and PD-weighted images of the pre- and post-stimulation scans were coregistered to the T1-weighted template. Following auto-reorienting to the AC-PC space, MT (MTsat), R1, PD and R2* maps were generated following published procedures (Helms, Dathe, & Dechent, 2008; Helms, Dathe, Kallenberg, et al., 2008;

Weiskopf et al., 2013, 2014). All maps were manually inspected for head movement and other image artifacts (data for 3 participants in the Anodal and 3 in the Sham group were removed from further analyses due to excessive head movement).

MPC and microstructural gradients. We used FreeSurfer (V6.0.0) recon-all command to perform cortical reconstruction and volumetric segmentation for MPM R1 maps. We used T1w maps for 5 participants as their surface reconstruction failed for R1 maps. We followed the same pipeline to compute MPC and to generate MPC gradients as for the multi-session training study.

Resting-state fMRI and FC gradients. We followed the same pipeline to compute FC gradients as for the multi-session training study.

Parcellation approach and principal gradient dispersion. Same methods were applied, as for the multi-session training study

Microarray gene expression from Allen Human Brain Atlas

We downsampled the data and selected the genes that are consistently expressed across the six donors following published recommendations (Arnatkevičiūtė et al., 2019), leaving 16651 genes in total (Wang et al., 2023). In brief, the expression data were parcellated to the Schäefer-200 atlas. Then, we set intensity-based filter = 50%, distance threshold = 2mm, applied scaled robust sigmoid for sample normalisation, normalised gene expressions to the same mean expression level across all samples, and aggregated probes to genes by differential stability (Arnatkevičiūtė et al., 2019; Markello et al., 2021). Median gene expression profiles for all genes were calculated for each node for further analysis.

Sensitivity analyses

Gene expression enrichment correction methods. We applied different types of correction methods (i.e. Benjamini, Fisher Exact, FDR, and Bonferroni) for gene expression enrichment analysis. The results remained similar across correction methods (Table S5).

Schaefer parcellations. We tested whether the results were consistent when using different Schaefer parcellations to calculate predictors (i.e. FPN within-network FC dispersion, VN-FPN between-network MPC dispersion) of learning rate. We calculated dispersion using 3 different atlases (Schäefer 200, 300, 400) and generated correlation matrices across atlases. Our results show that FPN within-network FC dispersions and VN-FPN between-network MPC dispersions are highly correlated across different Schäefer atlases (Figure S2) and remain significant predictors of learning rate (Table S6). Further, we conducted leave-one-out cross-validation on the regression analyses for a) FC dispersion (Table S7), b) MPC dispersion predicting learning rate (Table S8). The results remain significant, suggesting higher functional segregation of regions within FPN network and higher coherence in structural organization between FPN and EV networks after training relates to learning.

Supplementary Figures

Figure S1: 3D illustration of FC within-network dispersion in the fronto-parietal network (FPN). Red dots, blue dots and the shaded areas represent the dispersion of principal gradients for pre- and post-training sessions, respectively. The shaded area for the post-training session (blue) is larger than that for the pre-training session (red), indicating that the nodes representing functional connectivity similarity within the FPN network are more spread-out after training (i.e. higher network segregation).

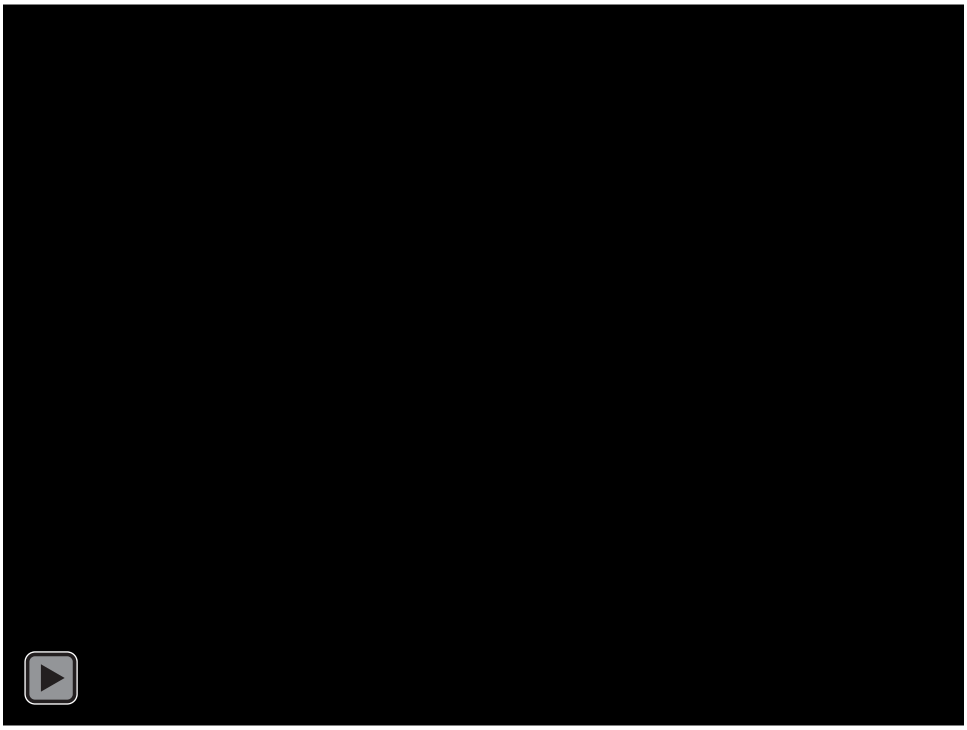

Figure S2. Correlation matrices across 3 different atlases (Schäefer 200, 300, 400). A. Correlation matrices of FPN within-network FC dispersion changes across atlases (post-training minus pre-training). B. Correlation matrices of EV-FPN between-network MPC dispersion changes across atlases (post-training minus pre-training). Correlation coefficients shown are between pairs of atlases.

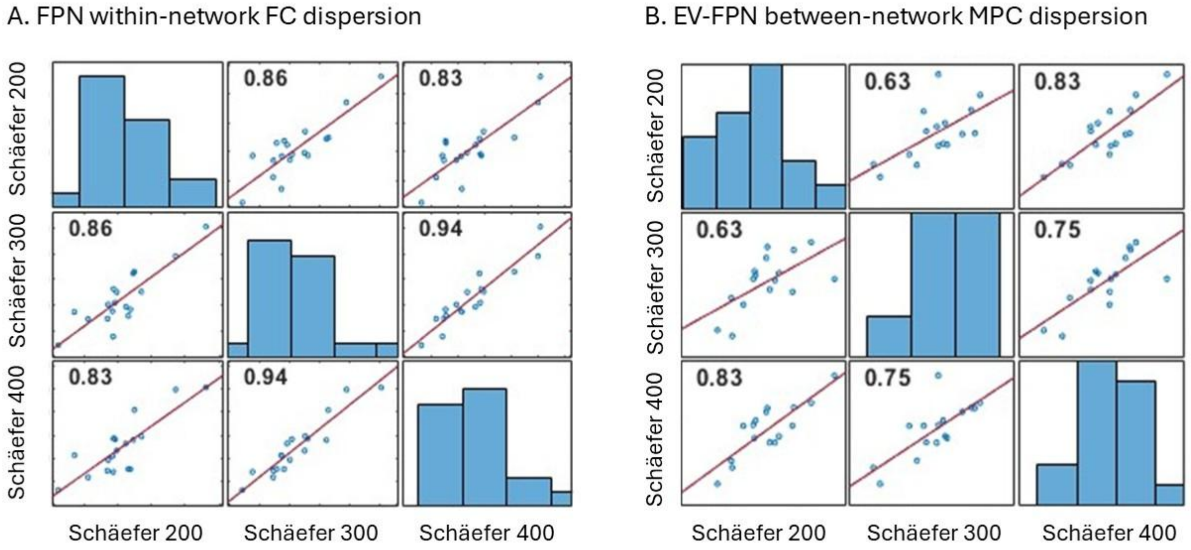

Supplementary Tables

Table S1. Top ranked 200 genes after FDR correction ($p < 0.05$)

rank	gene	weight (z-score)	rank	gene	weight (z-score)
1	PEA15	7.89	101	DENND1B	5.58
2	LPCAT4	7.44	102	DMKN	5.57
3	SYCP2	7.26	103	SV2A	5.57
4	ASB13	7.26	104	KDM7A	5.57
5	L2HGDH	7.26	105	HIVEP2	5.56
6	CCDC90B	7.12	106	SEMA7A	5.52
7	GPLD1	7.11	107	OST4	5.51
8	LINC00599	7.06	108	STARD5	5.51
9	RIPOR2	7.05	109	PPP3CC	5.50
10	ST3GAL6	6.98	110	KLHDC8B	5.49
11	UPP1	6.97	111	ZNF385B	5.48
12	HS3ST1	6.91	112	GABRA5	5.48
13	CPAMD8	6.84	113	CNOT6L	5.47
14	DPYSL3	6.83	114	GPM6B	5.46
15	NKAIN3	6.78	115	LRRC37A4P	5.46
16	LAG3	6.78	116	NR1D2	5.45
17	TTC21B	6.62	117	LUZP1	5.44
18	SLC4A8	6.57	118	CDH7	5.43
19	CXorf57	6.46	119	STUM	5.40
20	MIR29B2CHG	6.46	120	PCSK5	5.39
21	PRO1804	6.46	121	MTRF1	5.39
22	SOCS5	6.46	122	SPTSSB	5.37
23	DKFZp779M0652	6.45	123	SPAG4	5.36
24	INTS4P1	6.45	124	BID	5.36
25	LINC01637	6.40	125	ZBED9	5.36
26	CKMT1B	6.39	126	KCNAB3	5.35
27	STS	6.38	127	ALDH1A3	5.35
28	DDAH2	6.36	128	WDR86	5.35
29	WDR66	6.36	129	SLA	5.34
30	SLC25A12	6.36	130	TG	5.34
31	CACNA1H	6.34	131	DCBLD2	5.34
32	NCOA3	6.30	132	GLS2	5.30
33	TTC39B	6.30	133	LSM3	5.29
34	ESRRG	6.30	134	VPS36	5.29
35	PRKCD	6.28	135	FANCI	5.29
36	MMD	6.26	136	PCDH17	5.29
37	HES1	6.25	137	ATP2B4	5.29
38	PLPPR3	6.22	138	TRMT9B	5.27
39	SRPK1	6.21	139	HIST1H2BK	5.27
40	NTSR1	6.20	140	PRIM1	5.27

41	SNRNP27	6.18	141	GPR158	5.27
42	PDIK1L	6.17	142	TRIM37	5.27
43	TMEM263	6.16	143	ZADH2	5.25
44	NKAIN4	6.13	144	INTS8	5.25
45	CCDC58	6.13	145	TAF4B	5.24
46	CD63	6.11	146	SMC2	5.23
47	PRSS35	6.11	147	METTL24	5.21
48	GPCPD1	6.11	148	DPY19L2P2	5.20
49	GPAT3	6.11	149	TMEFF2	5.20
50	DPY19L2P1	6.11	150	RASGEF1C	5.19
51	SARNP	6.10	151	LUZP2	5.19
52	ENTPD4	6.10	152	LRRC49	5.18
53	FASTKD1	6.10	153	TIFA	5.18
54	OSBPL6	6.09	154	ZBTB1	5.17
55	TNFRSF25	6.08	155	C9orf72	5.17
56	MID2	6.08	156	MBNL3	5.16
57	TFB2M	6.05	157	IPW	5.16
58	IDE	6.04	158	MBOAT2	5.15
59	TRMT61B	6.02	159	FAM181A	5.15
60	FRAT1	6.00	160	SIRT4	5.15
61	LOC100506124	6.00	161	ELOVL4	5.15
62	THAP10	6.00	162	DNAJC4	5.15
63	AHI1	6.00	163	ZNF284	5.14
64	RPGR	5.98	164	CPSF1	5.14
65	RAD54B	5.98	165	RHOC	5.14
66	GABARAP	5.97	166	ZYX	5.14
67	KBTBD3	5.94	167	PPARGC1A	5.14
68	LIX1	5.94	168	XKR6	5.14
69	DCUN1D2	5.93	169	GNG2	5.14
70	C2CD4C	5.93	170	LRPPRC	5.14
71	SCN1B	5.92	171	OSBP2	5.13
72	SST	5.90	172	MYL12B	5.13
73	CASP8AP2	5.89	173	ANKH	5.13
74	SMARCD3	5.89	174	SUSD1	5.13
75	PIK3CA	5.85	175	UCHL5	5.13
76	SCN1A	5.85	176	SCRT1	5.12
77	KCNN3	5.76	177	TUBB6	5.12
78	SNHG14	5.75	178	BIRC3	5.11
79	KLF9	5.75	179	CXCR4	5.10
80	TMEM200A	5.72	180	ARMT1	5.10
81	HEBP1	5.72	181	SERTAD4	5.10
82	RORA	5.72	182	CCDC47	5.10
83	TFAM	5.72	183	CEP192	5.09
84	IFNLR1	5.68	184	SLIT1	5.09
85	CREM	5.68	185	CKS1B	5.08
86	ANK1	5.67	186	PANX2	5.08

87	FAM216A	5.67	187	LMO3	5.07
88	SLITRK3	5.65	188	MTPAP	5.07
89	FAM43A	5.64	189	TMED10	5.05
90	FNDC5	5.62	190	TCEA3	5.05
91	GFM1	5.62	191	FAM229B	5.05
92	ICA1	5.61	192	SIL1	5.05
93	TBC1D30	5.61	193	RNF113A	5.05
94	NKAIN2	5.61	194	RFX5	5.05
95	FAM71F1	5.59	195	NUDT11	5.05
96	BBS7	5.59	196	AMIGO2	5.04
97	NDRG3	5.58	197	TIGD3	5.04
98	EML6	5.58	198	RUNDC3B	5.03
99	FKBP1A	5.58	199	HSPA4L	5.03
100	DEFB131A	5.58	200	FOCAD	5.03

Table S2. Gene enrichment test for tissue types among brain tissues

Tissues	P-Value	Benjamin corrected
**Occipital Lobe**	**1.70E-08**	**4.50E-07**
**Prefrontal Cortex**	**5.90E-03**	**2.30E-02**
Amygdala	2.10E-01	5.40E-01
TemporalLobe	4.00E-01	8.50E-01
Cingulate Cortex	5.20E-01	1.00E+00
Hypothalamus	7.20E-01	1.00E+00
Thalamus	8.10E-01	1.00E+00
Parietal Lobe	8.40E-01	1.00E+00
Caudatenucleus	8.50E-01	1.00E+00
Whole Brain	8.90E-01	1.00E+00
Cerebellum	9.80E-01	1.00E+00

Table S3. Gene enrichment test for tissue types with null brain models and raw gene expressions

Tissues	P-Value	Benjamin corrected
**Occipital Lobe**	**1.30E-07**	**3.30E-06**
**Prefrontal Cortex**	**9.80E-03**	**4.00E-02**
Amygdala	2.10E-01	5.20E-01
TemporalLobe	3.40E-01	7.10E-01
Cingulate Cortex	4.10E-01	8.20E-01
Hypothalamus	7.70E-01	1.00E+00
Thalamus	8.20E-01	1.00E+00
Parietal Lobe	7.80E-01	1.00E+00
Caudatenucleus	8.50E-01	1.00E+00
Whole Brain	8.00E-01	1.00E+00
Cerebellum	9.90E-01	1.00E+00

Table S4. Gene enrichment test for tissue types with null brain models and denoised gene expressions

Tissues	P-Value	Benjamin corrected
**Occipital Lobe**	**5.50E-13**	**4.00E-11**
**Prefrontal Cortex**	**2.80E-05**	**1.70E-04**
Amygdala	7.00E-01	1.00E+00
TemporalLobe	1.80E-01	5.60E-01
Cingulate Cortex	2.40E-01	6.80E-01
Hypothalamus	9.00E-01	1.00E+00
Thalamus	6.20E-01	1.00E+00
Parietal Lobe	5.50E-01	1.00E+00
Caudatenucleus	1.00E+00	1.00E+00
Whole Brain	6.40E-01	1.00E+00
Cerebellum	8.10E-01	1.00E+00

Table S5. Gene expression enrichment with different correction methods.

**Tissues**	**P-Value**	**Benjamin**	**Fisher Exact**	**FDR**	**Bonferroni**
**Occipital Lobe**	**1.70E-08**	**4.50E-07**	**6.30E-18**	**9.60E-17**	**7.00E-16**
**Prefrontal Cortex**	**5.90E-03**	**2.30E-02**	**1.30E-03**	**4.70E-03**	1.20E-01
Amygdala	2.10E-01	5.40E-01	5.90E-01	8.30E-01	1.00E+00
Temporal Lobe	4.00E-01	8.50E-01	1.00E+00	8.30E-01	1.00E+00
Cingulate Cortex	5.20E-01	1.00E+00	9.50E-01	9.60E-01	1.00E+00
Hypothalamus	7.20E-01	1.00E+00	8.10E-01	8.30E-01	1.00E+00
Thalamus	8.10E-01	1.00E+00	6.90E-01	8.30E-01	1.00E+00
Parietal Lobe	8.40E-01	1.00E+00	7.50E-01	8.30E-01	1.00E+00
Caudatenucleus	8.50E-01	1.00E+00	3.40E-01	7.90E-01	1.00E+00
Whole Brain	8.90E-01	1.00E+00	4.70E-01	1.00E+00	1.00E+00
Cerebellum	9.80E-01	1.00E+00	1.00E+00	1.00E+00	1.00E+00

Table S6. Correlations of FPN within-network FC dispersion and VN-FPN between-network MPC dispersion with learning rate. Dispersion values are derived from different Schäefer atlases.

	**FPN within-network FC dispersion**		**VN-FPN between-network MPC dispersion**	
**Schäefer atlas**	**p**	**R**	**p**	**R**
**Schäefer 200**	0.002	0.668	0.019	-0.577
**Schäefer 300**	0.026	0.522	0.072	-0.461
**Schäefer 400**	0.034	0.501	0.013	-0.607

Table S7. Leave-one-out analysis for multiple regression analysis (FC dispersion to predict learning rate)

	**Model fit**			**Sig predictor (FPN within-network dispersion)**		
**Iteration**	**F**	**p**	$R^2_{Adjusted}$	**p**	**Beta**	**t**
1	8.139	0.003	0.572	0.028	1.136	4.596
2	7.594	0.003	0.553	<.001	1.080	4.314
3	7.843	0.003	0.562	<.001	1.100	4.386
4	7.697	0.003	0.557	<.001	0.942	4.277
5	7.132	0.004	0.535	0.001	1.056	4.167
6	6.908	0.005	0.526	0.002	1.005	3.825
7	6.943	0.005	0.527	0.001	1.070	4.122
8	7.022	0.005	0.530	0.002	0.932	3.964
9	6.799	0.005	0.521	0.002	1.041	3.978
10	6.565	0.006	0.511	0.002	1.039	3.961
11	6.596	0.006	0.512	0.003	0.988	3.631

12	6.558	0.006	0.510	0.001	1.040	4.005
13	6.637	0.006	0.514	0.001	1.070	4.138
14	6.598	0.006	0.512	0.001	1.039	4.006
15	6.750	0.006	0.519	0.001	1.065	4.039
16	6.456	0.007	0.506	0.002	1.044	3.957
17	5.378	0.013	0.451	0.002	1.033	3.744
18	5.120	0.015	0.436	0.004	1.148	3.504
mean	6.819	0.006	0.520	0.004	1.046	4.034
stdev.	0.755	0.003	0.034	0.007	0.056	0.266

Table S8. Leave-one-out analysis for multiple regression analysis (MPC dispersion to predict learning rate)

	**Model fit**			**Sig predictor (MPC between EV-FPN network dispersion)**		
**Iteration**	**F**	**p**	**$R^2_{Adjusted}$**	**p**	**Beta**	**t**
1	8.484	0.003	0.616	<.001	-0.843	-4.481
2	5.305	0.017	0.48	0.004	-0.802	-3.668
3	5.23	0.017	0.475	0.003	-0.81	-3.708
4	4.811	0.022	0.45	0.006	-0.796	-3.988
5	4.402	0.029	0.422	0.009	-0.731	-3.171
6	4.092	0.035	0.399	0.019	-0.662	-3.578
7	4.051	0.036	0.395	0.011	-0.725	-3.076
8	4.011	0.037	0.522	0.011	-0.723	-3.066
9	4.022	0.037	0.393	0.011	-0.72	-3.035
10	3.764	0.044	0.372	0.032	-0.548	-2.448
11	3.703	0.046	0.367	0.013	-0.71	-3.829
12	3.681	0.047	0.365	0.027	-0.671	-2.556
13	3.646	0.048	0.362	0.014	-0.651	-2.908
14	3.525	0.052	0.351	0.011	-0.749	-3.052
15	3.057	0.074	0.306	0.031	-0.697	-3.777
16	2.815	0.089	0.28	0.021	-0.689	-2.699
mean	4.287	0.040	0.410	0.015	-0.720	-3.315
stdev.	1.309	0.021	0.084	0.009	0.072	0.566

References

Arnatkevičiūtė, A., Fulcher, B. D., & Fornito, A. (2019). A practical guide to linking brain-wide gene expression and neuroimaging data. *NeuroImage*, *189*, 353–367. https://doi.org/10.1016/j.neuroimage.2019.01.011

Bethlehem, R. A. I., & Kitzbichler, M. G. (2023). *ucam-department-of-psychiatry/maps_and_parcs: V2 (Version V2)*. Zenodo. https://doi.org/https://doi.org/10.5281/zenodo.8116352

Bethlehem, R. A. I., Paquola, C., Seidlitz, J., Ronan, L., Bernhardt, B., Consortium, C.-C., & Tsvetanov, K. A. (2020). Dispersion of functional gradients across the adult lifespan. *NeuroImage*, *222*, 117299. https://doi.org/10.1016/j.neuroimage.2020.117299

Callaghan, M. F., Lutti, A., Ashburner, J., Balteau, E., Corbin, N., Draganski, B., Helms, G., Kherif, F., Leutritz, T., Mohammadi, S., Phillips, C., Reimer, E., Ruthotto, L., Seif, M., Tabelow, K., Ziegler, G., & Weiskopf, N. (2019). Example dataset for the hMRI toolbox. *Data in Brief*, *25*, 104132. https://doi.org/10.1016/j.dib.2019.104132

Cox, R. W. (1996). AFNI: Software for Analysis and Visualization of Functional Magnetic Resonance Neuroimages. *Computers and Biomedical Research*, *29*(3), 162–173. https://doi.org/10.1006/cbmr.1996.0014

Cross, N., Paquola, C., Pomares, F. B., Perrault, A. A., Jegou, A., Nguyen, A., Aydin, U., Bernhardt, B. C., Grova, C., & Dang-Vu, T. T. (2021). Cortical gradients of functional connectivity are robust to state-dependent changes following sleep deprivation. *NeuroImage*, *226*, 117547. https://doi.org/10.1016/j.neuroimage.2020.117547

Cruces, R. R., Royer, J., Herholz, P., Larivière, S., Vos de Wael, R., Paquola, C., Benkarim, O., Park, B., Degré-Pelletier, J., Nelson, M. C., DeKraker, J., Leppert, I. R., Tardif, C., Poline, J.-B., Concha, L., & Bernhardt, B. C. (2022). Micapipe: A pipeline for multimodal neuroimaging and connectome analysis. *NeuroImage*, *263*, 119612. https://doi.org/10.1016/j.neuroimage.2022.119612

Fischl, B. (2012). FreeSurfer. *NeuroImage*, *62*(2), 774–781. https://doi.org/10.1016/j.neuroimage.2012.01.021

Frangou, P., Correia, M., & Kourtzi, Z. (2018). GABA, not BOLD, reveals dissociable learning-dependent plasticity mechanisms in the human brain. *ELife*, *7*. https://doi.org/10.7554/eLife.35854

Frangou, P., Emir, U. E., Karlaftis, V. M., Nettekoven, C., Hinson, E. L., Larcombe, S., Bridge, H., Stagg, C. J., & Kourtzi, Z. (2019). Learning to optimize perceptual decisions through suppressive interactions in the human brain. *Nature Communications*, *10*(1), 474. https://doi.org/10.1038/s41467-019-08313-y

Glass, L. (1969). Moiré Effect from Random Dots. *Nature*, *223*(5206), 578–580. https://doi.org/10.1038/223578a0

Greve, D. N., & Fischl, B. (2009). Accurate and robust brain image alignment using boundary-based registration. *NeuroImage*, *48*(1), 63–72. https://doi.org/10.1016/j.neuroimage.2009.06.060

Helms, G., Dathe, H., & Dechent, P. (2008). Quantitative FLASH MRI at 3T using a rational approximation of the Ernst equation. *Magnetic Resonance in Medicine*, *59*(3), 667–672. https://doi.org/10.1002/mrm.21542

Helms, G., Dathe, H., Kallenberg, K., & Dechent, P. (2008). High-resolution maps of magnetization transfer with inherent correction for RF inhomogeneity and T_1 relaxation obtained from 3D FLASH MRI. *Magnetic Resonance in Medicine*, *60*(6), 1396–1407. https://doi.org/10.1002/mrm.21732

Jenkinson, M., Beckmann, C. F., Behrens, T. E. J., Woolrich, M. W., & Smith, S. M. (2012). FSL. *NeuroImage*, *62*(2), 782–790. https://doi.org/10.1016/j.neuroimage.2011.09.015

Marcus, D. S., Harwell, J., Olsen, T., Hodge, M., Glasser, M. F., Prior, F., Jenkinson, M., Laumann, T., Curtiss, S. W., & Van Essen, D. C. (2011). Informatics and Data Mining Tools and Strategies for the Human Connectome Project. *Frontiers in Neuroinformatics*, *5*. https://doi.org/10.3389/fninf.2011.00004

Markello, R. D., Arnatkeviciute, A., Poline, J.-B., Fulcher, B. D., Fornito, A., & Misic, B. (2021). Standardizing workflows in imaging transcriptomics with the abagen toolbox. *ELife*, *10*. https://doi.org/10.7554/eLife.72129

Mayhew, S. D., Li, S., & Kourtzi, Z. (2012). Learning Acts on Distinct Processes for Visual Form Perception in the Human Brain. *The Journal of Neuroscience*, *32*(3), 775–786. https://doi.org/10.1523/JNEUROSCI.2033-11.2012

O'Shea, J., Revol, P., Cousijn, H., Near, J., Petitet, P., Jacquin-Courtois, S., Johansen-Berg, H., Rode, G., & Rossetti, Y. (2017). Induced sensorimotor cortex plasticity remediates chronic treatment-resistant visual neglect. *ELife*, *6*. https://doi.org/10.7554/eLife.26602

Schaefer, A., Kong, R., Gordon, E. M., Laumann, T. O., Zuo, X.-N., Holmes, A. J., Eickhoff, S. B., & Yeo, B. T. T. (2018). Local-Global Parcellation of the Human Cerebral Cortex from Intrinsic Functional Connectivity MRI. *Cerebral Cortex*, *28*(9), 3095–3114. https://doi.org/10.1093/cercor/bhx179

Stagg, C. J., Jayaram, G., Pastor, D., Kincses, Z. T., Matthews, P. M., & Johansen-Berg, H. (2011). Polarity and timing-dependent effects of transcranial direct current stimulation in explicit motor learning. *Neuropsychologia*, *49*(5), 800–804. https://doi.org/10.1016/j.neuropsychologia.2011.02.009

Stanislaw, H., & Todorov, N. (1999). Calculation of signal detection theory measures. *Behavior Research Methods, Instruments, & Computers*, *31*(1), 137–149. https://doi.org/10.3758/BF03207704

Tabelow, K., Balteau, E., Ashburner, J., Callaghan, M. F., Draganski, B., Helms, G., Kherif, F., Leutritz, T., Lutti, A., Phillips, C., Reimer, E., Ruthotto, L., Seif, M., Weiskopf, N., Ziegler, G., & Mohammadi, S. (2019). hMRI – A toolbox for quantitative MRI in neuroscience and clinical research. *NeuroImage*, *194*, 191–210. https://doi.org/10.1016/j.neuroimage.2019.01.029

Thomas, C., & Baker, C. I. (2013). Teaching an adult brain new tricks: A critical review of evidence for training-dependent structural plasticity in humans. *NeuroImage*, *73*, 225–236. https://doi.org/10.1016/j.neuroimage.2012.03.069

Vos de Wael, R., Benkarim, O., Paquola, C., Lariviere, S., Royer, J., Tavakol, S., Xu, T., Hong, S.-J., Langs, G., Valk, S., Misic, B., Milham, M., Margulies, D., Smallwood, J., & Bernhardt, B. C. (2020). BrainSpace: a toolbox for the analysis of macroscale gradients in neuroimaging and connectomics datasets. *Communications Biology*, *3*(1), 103. https://doi.org/10.1038/s42003-020-0794-7

Waehnert, M. D., Dinse, J., Weiss, M., Streicher, M. N., Waehnert, P., Geyer, S., Turner, R., & Bazin, P.-L. (2014). Anatomically motivated modeling of cortical laminae. *NeuroImage*, *93*, 210–220. https://doi.org/10.1016/j.neuroimage.2013.03.078

Wagstyl, K., Lepage, C., Bludau, S., Zilles, K., Fletcher, P. C., Amunts, K., & Evans, A. C. (2018). Mapping Cortical Laminar Structure in the 3D BigBrain. *Cerebral Cortex*, *28*(7), 2551–2562. https://doi.org/10.1093/cercor/bhy074

Wang, Y., Royer, J., Park, B., Vos de Wael, R., Larivière, S., Tavakol, S., Rodriguez-Cruces, R., Paquola, C., Hong, S.-J., Margulies, D. S., Smallwood, J., Valk, S. L., Evans, A. C., & Bernhardt, B. C. (2023). Long-range functional connections mirror and link microarchitectural and cognitive hierarchies in the human brain. *Cerebral Cortex*, *33*(5), 1782–1798. https://doi.org/10.1093/cercor/bhac172

Weiskopf, N., Callaghan, M. F., Josephs, O., Lutti, A., & Mohammadi, S. (2014). Estimating the apparent transverse relaxation time (R2*) from images with different contrasts (ESTATICS) reduces motion artifacts. *Frontiers in Neuroscience*, *8*. https://doi.org/10.3389/fnins.2014.00278

Weiskopf, N., Suckling, J., Williams, G., Correia, M. M., Inkster, B., Tait, R., Ooi, C., Bullmore, E. T., & Lutti, A. (2013). Quantitative multi-parameter mapping of R1, PD*, MT, and R2* at 3T: a multi-center validation. *Frontiers in Neuroscience*, *7*. https://doi.org/10.3389/fnins.2013.00095

Yeo, B. T. T., Krienen, F. M., Sepulcre, J., Sabuncu, M. R., Lashkari, D., Hollinshead, M., Roffman, J. L., Smoller, J. W., Zöllei, L., Polimeni, J. R., Fischl, B., Liu, H., & Buckner, R. L. (2011). The organization of the human cerebral cortex estimated by intrinsic functional connectivity. *Journal of Neurophysiology*, *106*(3), 1125–1165. https://doi.org/10.1152/jn.00338.2011

Ziminski, J. J., Frangou, P., Karlaftis, V. M., Emir, U., & Kourtzi, Z. (2023). Microstructural and neurochemical plasticity mechanisms interact to enhance human perceptual decision-making. *PLOS Biology, 21*(3), e3002029. https://doi.org/10.1371/journal.pbio.3002029
