# Supplementary material for: Neurogenetic phenotypes of learning-dependent plasticity for improved perceptual decisions
Source: Commun Biol. 2025 May 21;8:779. doi: 10.1038/s42003-025-08212-7 (PMC12095785; doi:10.1038/s42003-025-08212-7)
Supplement: Supplementary file 3 — reporting summary [file 42003_2025_8212_MOESM3_ESM.pdf]

nature portfolio

Corresponding author(s): Zoe Kourtzi
Last updated by author(s): 02/05/2025

Reporting Summary

Nature Portfolio wishes to improve the reproducibility of the work that we publish. This form provides structure for consistency and transparency in reporting. For further information on Nature Portfolio policies, see our Editorial Policies and the Editorial Policy Checklist.

Statistics

For all statistical analyses, confirm that the following items are present in the figure legend, table legend, main text, or Methods section.

n/a	Confirmed	
☐	☒	The exact sample size (*n*) for each experimental group/condition, given as a discrete number and unit of measurement
☐	☒	A statement on whether measurements were taken from distinct samples or whether the same sample was measured repeatedly
☐	☒	The statistical test(s) used AND whether they are one- or two-sided<br>*Only common tests should be described solely by name; describe more complex techniques in the Methods section.*
☐	☒	A description of all covariates tested
☐	☒	A description of any assumptions or corrections, such as tests of normality and adjustment for multiple comparisons
☐	☒	A full description of the statistical parameters including central tendency (e.g. means) or other basic estimates (e.g. regression coefficient) AND variation (e.g. standard deviation) or associated estimates of uncertainty (e.g. confidence intervals)
☐	☒	For null hypothesis testing, the test statistic (e.g. *F*, *t*, *r*) with confidence intervals, effect sizes, degrees of freedom and *P* value noted<br>*Give P values as exact values whenever suitable.*
☒	☐	For Bayesian analysis, information on the choice of priors and Markov chain Monte Carlo settings
☒	☐	For hierarchical and complex designs, identification of the appropriate level for tests and full reporting of outcomes
☐	☒	Estimates of effect sizes (e.g. Cohen's *d*, Pearson's *r*), indicating how they were calculated

Our web collection on statistics for biologists contains articles on many of the points above.

Software and code

Policy information about availability of computer code

Data collection	Sequence and code used in the study is available in the Cambridge University repository (https://doi.org/10.17863/CAM.117105).
Data analysis	Code used in the study is available in the Cambridge University repository (https://doi.org/10.17863/CAM.117105).

For manuscripts utilizing custom algorithms or software that are central to the research but not yet described in published literature, software must be made available to editors and reviewers. We strongly encourage code deposition in a community repository (e.g. GitHub). See the Nature Portfolio guidelines for submitting code & software for further information.

Data

Policy information about availability of data

All manuscripts must include a data availability statement. This statement should provide the following information, where applicable:

- Accession codes, unique identifiers, or web links for publicly available datasets
- A description of any restrictions on data availability
- For clinical datasets or third party data, please ensure that the statement adheres to our policy

The anonymised data collected in the study will be available in the Cambridge University repository (https://doi.org/10.17863/CAM.117105).

Human research participants

Policy information about studies involving human research participants and Sex and Gender in Research.

Reporting on sex and gender	For the multi-session training study, only data from male participants was included. The data was collected as part of a previous study, which included MRS data collection. The study sample was limited to male participants due to potential confounding effects of menstrual cycle on GABA measurements. This has been the topic of extensive research with several studies restricting MRS-GABA studies to males. Key hormones (estrogen, progesterone) exerting a suppressive or facilitatory effect on GABA transmission may confound within-subject GABA measurements over time. Developing precise methods for controlling for the effects of menstrual cycle on MRS GABA measurements is hampered by physiological complexity (i.e., phase and regional effects of menstrual cycle on GABA) and limited knowledge of the kinetics of menstrual cycle GABA changes in humans. As the study involves repeated MRS GABA measurements over time, it is not possible to satisfactorily control for menstrual cycle effects; i.e., not only the phase and duration of the menstrual cycle but also the kinetics of GABA changes across menstrual cycle days would likely differ substantially across participants.<br><br>For the tDCS and training intervention study, data was collected from both male and female participants.
Population characteristics	Healthy volunteers. In the multi-session training study, Twenty-two healthy volunteers (23.5 ± 4.2 years) participated. In the tDCS and training intervention study: forty-five healthy volunteers (mean age 22.9 ± 3.3 years)
Recruitment	Participants were recruited from Cambridge university via posters and online forums.
Ethics oversight	University of Cambridge Ethics Committee

Note that full information on the approval of the study protocol must also be provided in the manuscript.

Field-specific reporting

Please select the one below that is the best fit for your research. If you are not sure, read the appropriate sections before making your selection.

☒ Life sciences ☐ Behavioural & social sciences ☐ Ecological, evolutionary & environmental sciences

For a reference copy of the document with all sections, see nature.com/documents/nr-reporting-summary-flat.pdf

Life sciences study design

All studies must disclose on these points even when the disclosure is negative.

Sample size	In the multi-session training study, the sample size is comparatable to other studies. in the tDCS and training intervention study, sample size was guided by power calculations.
Data exclusions	Data from 2 participants were excluded because they did not show improvement in the behavioral task, as defined by a positive learning rate across sessions. Five participants were excluded from MPM analyses due to poor map quality as assessed by MRI data quality.
Replication	Both behavioural and imaging results replicated previous studies (Frangou et al., 2019, eLife).
Randomization	In the tDCS and training intervention study, participants were randomly assigned to groups.
Blinding	The researchers were blinded to the groups during participants allocation and data analysis.

Reporting for specific materials, systems and methods

We require information from authors about some types of materials, experimental systems and methods used in many studies. Here, indicate whether each material, system or method listed is relevant to your study. If you are not sure if a list item applies to your research, read the appropriate section before selecting a response.

Materials & experimental systems

n/a	Involved in the study
☒	☐ Antibodies
☒	☐ Eukaryotic cell lines
☒	☐ Palaeontology and archaeology
☒	☐ Animals and other organisms
☒	☐ Clinical data
☒	☐ Dual use research of concern

Methods

n/a	Involved in the study
☒	☐ ChIP-seq
☒	☐ Flow cytometry
☐	☒ MRI-based neuroimaging

Magnetic resonance imaging

Experimental design

Design type

multi-session training study: resting-state fMRI and microstructure changes pre- and post-training.
tDCS and training intervention study: resting-state fMRI and microstructure changes pre- and post-training, anodal and sham stimulation between-group comparison.

Design specifications

In the multi-session training study: Each volunteer participated in six sessions: three brain-imaging sessions including testing on the SN task without feedback (day 1: baseline, day 5: pre-training, day 9: post-training) and three consecutive task training behavioral sessions with feedback (day 6, day 7, day 8). During each session, participants completed 8 runs (200 trials per run) of the SN tasks. Each run comprised an equal number of trials presenting concentric and radial patterns. In each trial, a fixation dot appeared for 500-1500ms, followed by a radial or concentric pattern for 300 ms. Participants were asked to judge whether the stimulus presented was radial or concentric. Trial-by-trial feedback was provided by means of a visual cue (green tick for correct, red “x” for incorrect), which remained on the screen for 200 ms and was followed by a fixation dot for a variable time between 500 and 1,500 ms before the next trial onset. Measurements pre- and post-training were compared. In the tDCS and training intervention study, participants completed 9 blocks of 200 trials. Measurements between stimulation groups were compared.

Behavioral performance measures

multi-session training study: We calculated a) performance accuracy (i.e. percentage of correct responses) for each session, b) learning rate that indicates the rate of change in perceptual sensitivity as measured by d’ per training run. To compute learning rate, we fitted individual participant training data with a logarithmic function: y = k * ln x+ c, where x is the training run, y is the run d’, c is the starting d’ and k corresponds to the learning rate. Data from 2 participants were excluded because they did not show improvement in the behavioral task, as defined by a positive learning rate across sessions.

Acquisition

Imaging type(s)

Functional and structural (multi-parameter mapping)

Field strength

3 Tesla

Sequence & imaging parameters

Whole-brain MPM data were collected using a spoiled multi-echo 3D fast low-angle shot (FLASH) protocol of 3 gradient acquisitions: MT saturation, T1-weighted, and proton density (PD) weighted maps. All weighted maps had 0.8 mm isotropic resolution, field of view of 256 × 240 × 176 mm, and readout bandwidth of 488 Hz/pixel and were collected with partially parallel imaging in each phase-encoded (AP, RL) direction (GRAPPA, 40 integrated autocalibrating lines in each direction, acceleration factor of 2). We used a semiquantitative MT saturation (MTsat) sequence that accounts for spatially varying T1 and B1+, enhancing specificity to myelin content. For MT (excitation flip angle of 6°), we acquired 6 gradient echoes with alternating readout gradient polarity at echo times ranging from 2.30 to 18.40 ms in steps of 2.30 ms. For PD (excitation flip angle of 6°) and T1 (excitation flip angle of 21°), we acquired 8 gradient echoes with alternating readout gradient polarity at echo times ranging from 2.30 to 18.40 ms in steps of 2.30 ms. Unaccelerated 8 mm isotropic head and body coil sensitivity bias fields (TR: 6 ms, TE 2.20 ms, flip angle: 6°) were collected before each FLASH acquisition. To correct for field inhomogeneities and susceptibility distortions, we collected B1 and B0 fieldmaps. B1 field maps were acquired with 11 spin-echo and stimulated spin-echo pairs (TR: 500 ms, 4 mm isotropic resolution, 3D-EPI readout with 0.5 ms echo spacing, echo time 39.06 ms, mixing-time 33.8 m) with flip angle between 115˚to 65˚in 5˚ increments. B0 maps were acquired with a flip angle of 60˚(TR: 1020 ms, 3 × 3 × 2 mm resolution. Two images with echo times of 10.00 and 12.46 ms at 260 Hz/pixel bandwidth were acquired, and the phase difference image was generated by Siemens software.
Echo-planar imagining (EPI) acquisitions with full brain coverage (TR: 727 ms, TE: 34.6 ms, slices: 72; voxel size: 2 mm isotropic; multi-band factor: 8; flip angle 48º; volumes: 812) were collected for resting-state fMRI.

Area of acquisition

whole brain

Diffusion MRI ☐ Used ☒ Not used

Preprocessing

Preprocessing software

MATLAB hMRI toolbox to create MPM maps. FreeSurfer, micapipe toolbox, and Python BrainSpace package to generate functional and microstructral gradients.

Normalization	Imaging data were analysed in individual spaces with FreeSurfer and micapipe. We computed a group gradient template for both functional and microstructural gradients, and then individual gradients were aligned to the group template.
Normalization template	We computed a group gradient template for both functional and microstructural gradients, and then individual gradients were aligned to the group template.
Noise and artifact removal	FSL-based motion correction, Distortion correction, and Nuisance signal removal were performed (embedded in micapipe toolbox pipeline) for resting-state fMRI data. Five participants were excluded from MPM analyses due to poor map quality as assessed by a PD map error estimate less than 8% (mean / SD white matter intensity) and visual inspection.
Volume censoring	NA

Statistical modeling & inference

Model type and settings	We extracted features from imaging data and performed t-test and ANOVA analysis.
Effect(s) tested	multi-session training study: we extracted features from imaging data and compared mean values pre- and post-training with t-test.<br>tDCS and training intervention study: we extracted features from imaging data and compared mean values with two-way repeated measures ANOVA (significant Group (Anodal, Sham) x Block (Pre-, Post-stimulation)).

Specify type of analysis: ☐ Whole brain ☐ ROI-based ☒ Both

Anatomical location(s)	We used Schaefer 200, 300, 400 parcellation for whole brain analysis. For ROI-based analysis, Visual network and Frontoparietal networks were defined by Yeo 7 functional brain networks atlas.
Statistic type for inference (See Eklund et al. 2016)	NA. We extracted features from imaging data.
Correction	FDR correction was used for multiple comparison.

Models & analysis

n/a	Involved in the study
☐	☒ Functional and/or effective connectivity
☒	☐ Graph analysis
☒	☐ Multivariate modeling or predictive analysis

Functional and/or effective connectivity	We used functional connectivity and further generated FC gradient. Multiple regression, ANOVA, and t-test were performed for FC gradient analysis.
